# Cannabinol: History, Syntheses, and Biological Profile of the Greatest “Minor” Cannabinoid

**DOI:** 10.3390/plants11212896

**Published:** 2022-10-28

**Authors:** Chiara Maioli, Daiana Mattoteia, Hawraz Ibrahim M. Amin, Alberto Minassi, Diego Caprioglio

**Affiliations:** 1 Department of Pharmaceutical Sciences, University of Piemonte Orientale, Largo Guido Donegani 2/3, 28100 Novara, Italy; 2 The Armenise-Harvard Laboratory of Structural Biology, Department of Biology and Biotechnology, University of Pavia, 27100 Pavia, Italy; 3 PlantaChem SRLS, Via Canobio 4/6, 28100 Novara, Italy

**Keywords:** cannabinol, cannabinoids, phytocannabinoids, cannabinome

## Abstract

Cannabis (*Cannabis sativa* L.) is an outstanding source of bioactive natural products, with more than 150 different phytocannabinoids isolated throughout the decades; however, studies of their bioactivity have historically concentrated on the so-called “big four” [∆^9^-THC (**1a**), CBD (**2a**), CBG (**3a**) and CBC (**4a**)]. Among the remaining products, which have traditionally been referred to as “minor cannabinoids”, cannabinol (CBN, **5a**) stands out for its important repercussions and implications on the global scientific landscape. Throughout this review, we will describe why CBN (**5a**) deserves a prominent place within the so-called “cannabinome”, providing an overview on its history, the syntheses developed, and its bioactivity, highlighting its promising pharmacological potential and the significant impact that the study of its chemistry had on the development of new synthetic methodologies.

## 1. Introduction

There is extensive historical evidence that cannabis (*Cannabis sativa* L.) has been used for different purposes, among them industrial [1], ornamental [2], and pharmaceutical (e.g., treating rheumatic pain, constipation, gout, and gynecological disorders) [3] applications. Nowadays, the intake of marijuana is permitted in many countries of the world for the treatment of different pathologies [4], including nausea caused by chemotherapy, anorexia in patients suffering from AIDS, and pain management [5,6].

The biological activity of cannabis is strictly related to phytocannabinoids, the hallmark secondary metabolites of this remarkable plant [7]. This class of meroterpenoids is characterized by great chemical diversity among its constituents; however, a general phytocannabinoid structure is easily identifiable due to its characteristic hybrid nature; since this class derives from the merging of polyketides and amevalonate biosynthetic pathway, a resorcinyl core decorated with *p*-oriented isoprenyl residues and an alkyl side chain is easily identifiable [8].

**Figure 1:**
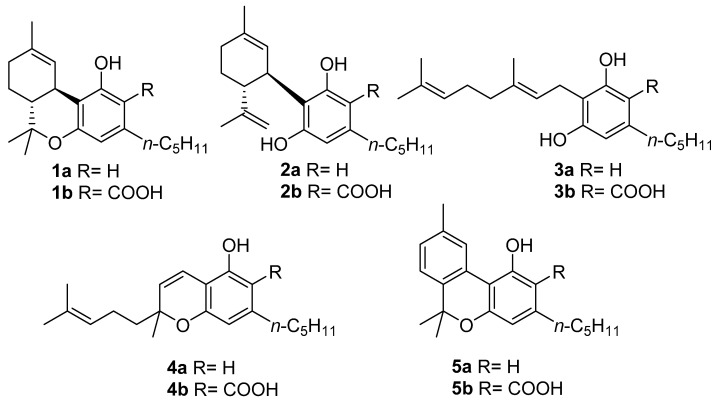
The structures of the “big four” phytocannabinoids [∆^9^-THC (**1a**), CBD (**2a**), CBG (**3a**) and CBC (**4a**)] and their acidic precursors (**1b**–**4b**), CBN (**5a**) and its acidic form CBNA (**5b**).

Despite this impressive variety, early studies in this field focused almost exclusively on the narcotic principle of marijuana, ∆^9^-tetrahydrocannabinol (∆^9^-THC, **1a**, Figure 1), eventually expanding to the other related compounds, which are cannabidiol (CBD, **2a**, Figure 1), cannabigerol (CBG, **3a**, Figure 1), and cannabichromene (CBC, **4a**, Figure 1), that together form a group of compounds often referred to as “the major cannabinoids” or “big four”[9]. As our understanding of the biological mechanisms underlying ∆^9^-THC narcotic properties developed, the endocannabinoid system (ECS) was discovered [10], and its complexity, homeostatic role, and potential for drug discovery prompted a reconsideration of the other three major cannabinoids derived from cannabis. In addition to these studies, CBD (**2a**) was developed into a standardized extract (Sativex) and as an active pharmaceutical ingredient (API) (Epidiolex). The latter is the drug of choice for the treatment of certain rare genetic forms of epilepsy, while Sativex (a combination of ∆^9^-THC and CBD) is used to treat spasticity associated with multiple sclerosis [5]. Likewise, CBG (**3a**) and CBC (**4a**) found their way into the literature as well [11,12].

Most of the studies on phytocannabinoids, both chemical and biological, have focused on the “big four” due to their high extraction yield from vegetable sources or easy accessibility through total synthesis [11,12,13,14,15,16,17]. However, cannabis plants are also capable of producing more than 150 other compounds referred to as “minor cannabinoids” [18], which have significant structural differences and specific biological properties [19,20]; among these compounds, one stands out in particular, namely cannabinol (CBN, **5a**, Figure 1).

CBN (**5a**) is one of the most famous phytocannabinoids in *C. sativa*, and although several phytocannabinoids have been identified in different plants and fungi, CBN (**5a**) has only been identified in cannabis. In the cannabinoid family, CBN (**5a**) is unique in several ways, the main one being its origin: whereas the acidic precursors of major cannabinoids (**1b**, **2b**,**4b**, Figure 1) are generated by the result of different cyclizations of the terpenyl moiety of cannabigerolic acid (CBGA, **3b**, in turn obtained from the condensation of olivetolic acid with geranylpyrophosphate) mediated by particular cyclases [21], a biosynthetic pathway for cannabinolic acid (CBNA, **5b**, Figure 1), and so of CBN (**5a**) itself, has not been identified; the latter is a degradation artifact of ∆^9^-THC (**1a**) which, as result of air oxidation, undergoes aromatization at the level of the menthyl moiety [22]. However, small amounts of CBNA (**5b**) have been found in some hemp samples [23], suggesting that, in particular conditions, oxidative degradation may also occur to tetrahydrocannabinolic acid (THCA, **1b**, Figure 1), the acidic precursor of ∆^9^-THC (**1a**), as well as before decarboxylation.

CBN (**5a**) was probably considered a “minor” phytocannabioid owing to its unfortunate and confusing discovery: it was the first phytocannabinoid to be isolated from hashish in the late 19th century, but its structure was not fully solved until 1940 due to some issues related to both nomenclature misunderstandings and the nature of the plant material used for the extraction (see Section 2). This confusing situation—associated with its limited availability and the discovery of more interesting bioactive phytocannabinoids—severely hindered its characterization from a biological and pharmacological standpoint.

Therefore, our purpose is to give this forgotten and mistreated phytocannabinoid due prominence and attention, focusing on its well-known synthetic pathways and highlighting the urgent need to fill the gaps in its biological field.

## 2. History

Marijuana is perhaps one of the oldest plants grown by mankind, so much that we can say that *Cannabis sativa* L. and humanity share a close and intertwined history [24]. From the perspective of time, CBN (**5a**) probably plays the most important role of all the cannabinoids: due to its exceptional stability and direct chemical relationship with the psychoactive constituent Δ^9^-THC (**1a**), the natural product has been assumed to be the most relevant marker [25] for the identification of narcotic cannabis in archaeological plant samples.

CBN’s (**5a**) extraordinary stability has been demonstrated by the discovery of plant material (seeds) dating back to 750 BC, found in a tomb in the Xinjiang-Uighur autonomous region (China), still containing high levels of the molecule [26].

Also attributed to CBN (**5a**) are the great uncertainty and ambiguity that plagued the first studies on the psychotropic properties of *C. sativa*: a significant limitation of the initial researches was related to the poor quality of the plant material investigated, which was mostly imported, directly or through Egypt, from India [27]. As a result of the long journey between the collection site and the European laboratories, usually lasting months, combined with uncontrolled and careless storage conditions, a sharp reduction in Δ^9^-THC (**1a**) levels in favor of CBN (**5a**) was achieved, leading to a relative falsification of the biological observations [28].

**Figure 2:**
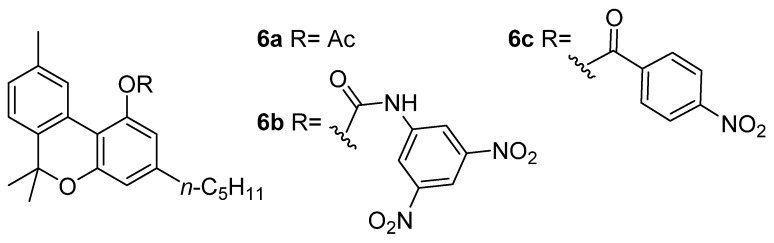
The structures of CBN adducts used for its isolation and characterization: CBN-acetate (**6a**), CBN-3,5-dinitrophenylurethane (**6b**) and CBN-4-nitrophenyl ester (**6c**).

Wood coined the term “cannabinol” at the end of the 19th century in order to describe the “red oil”, a dense resin containing both CBN (**5a**) and other major phytocannabinoids as well [29]; these compounds are brownish and colorless oils or white solids, whereas the ruby red color likely resulted from quinoid structures [30] forming during the oil purification. Obtaining this resin required a difficult and complex preparation optimized by Wood himself, Spivey, and Easterfield at Cambridge University, and entailed the distillation of ethanolic or ethereal Cannabis extract at reduced pressure (2 mm) and collecting the vapors at a temperature of between 100 and 220 °C (corresponding to a bath temperature of 170–300 °C) [29]. Initially considered to be a pure compound, “red oil” was acetylated by Easterfield resulting in a crystalline compound with an optically inactive nature (**6a**, Figure 2). Its natural phenol (**5a**) was then referred to as “cannabinol”, taking the name used previously for the narcotic red oil and making CBN (**5a**) the first phytocannabinoid isolated [31].

A tragic fate marked by CBN (**5a**) awaited the trio of scientists, despite their initial promising results: Spivey died in an explosion during the nitration of the “red oil” [32], while Wood became severely ill after voluntarily testing its effects [33]. Aside from this, Easterfield is also reported to have died during this research, due to an explosion associated with the hydrogenation of the “red oil”, though later sources report that he survived and relocated to New Zealand [34].

An additional intriguing, albeit tragic episode related to the first research on CBN (**5a**) involves another scientist of the same age, C. R. Marshall, who ingested approximately 100 mg of “red oil” as a means of combating boredom during the distillation of diethylzinc, a highly flammable liquid, to assess whether the hypothetic compound was narcotic. A little more than 45 min later, he was found leaning against the distillation flask in the lab, giggling and repeating “*this is lovely*” as flames spread around him due to oxygen leaks that set fire to diethylzinc. Only the prompt intervention of Marshall’s colleagues prevented a catastrophe, and he recovered quickly afterward [35]. Despite its historical significance, the “red oil” brought, in addition to the tragedies mentioned above, a great deal of confusion to the cannabis research community during its early years, mainly due to two major factors: first, the issue of the name “cannabinol” itself, which has been transferred from the distillate (narcotic) to the natural product (inactive) [31]; the second reason is related to the fact that the plant material from which the oil was distilled often exhibited a fluctuating phytochemical profile resulting from different methods of storage under ambiguous conditions, a profile further influenced by the dramatic conditions of the resin distillation [27].

A “full stop” to the confusion that hovered over CBN (**5a**) was posed in the 1930s by Cahn (famous for nomenclature and stereochemistry with the Cahn–Ingold–Prelog rules [36]): by defining red oil as “raw cannabinol” and the pure compound as “cannabinol”, he was able to elucidate almost completely the structure of the natural product through degradation studies, identifying a dibenzopyranic structure with the presence of a phenol and of a *n*-pentyl on the resorcinyl portion, indicating their relative positions [37].

In the following decade, two scientists worked independently on elucidating this structure, Roger Adams from Illinois State University and Alexander R. Todd (“Tod Almighty” to his students [38]) from the University of Manchester, obtaining several results that still have significant implications for the field of cannabinoids research. In his own experiments, the first obtained crystalline phenylurethane of CBN (**6b**) during a procedure involving 3,5-dinitrobenzoylazide treatment of “red oil” [39]; instead, the second sought to remove virtually all CBN from the mother liquor as *p*-nitrobenzoyl chloride ester (**6c**) [40], allowing him to isolate another cannabinoid, later identified as CBD (**2a**) [41]. Through a total synthesis of the CBN (**5a**) in 1940, Adams was able to establish the structure of the molecule definitively [42] (see Section 3.2).

Over the following decades, with the isolation and characterization of major phytocannabinoids, research on CBN (**5a**) was slowly set aside, eclipsed by the important and marked pharmacological activity of two compounds in particular, Δ^9^-THC (**1a**) and CBD (**2a**). The diphenyl structure of the natural compound, however, makes it an important model compound for the development of new synthetic methodologies that have led to the design of numerous total syntheses (see Section 3).

## 3. Synthesis

In today’s world, total synthesis has advanced dramatically thanks to powerful methodologies and sophisticated instruments; however, even for structures that may appear small and simple at first glance, the total synthesis of organic molecules still poses significant challenges for chemists, especially in terms of time efficiency, yields and, more importantly, environmentally friendly processes [43].

The need for simple and effective syntheses of CBN (**5a**) arises from the fact that extractions from plant sources are extremely limited for the following reasons: first, the yields are not very reproducible, since the concentration of the natural product is closely related to the state of conservation of the plant materials; in addition, CBN (**5a**) exhibits characteristics of polarity and solubility that are similar to those of other cannabinoids—primarily Δ^9^-THC (**1a**)—factors that make extraction a disadvantageous option. In addition to derivatization from “red oil” [29], currently new, more modern methods have been reported, primarily using long-lasting liquid extraction in a Soxhlet apparatus or pressurized liquid extraction [44], with ultrasonication of the extract reported to be an effective method for increasing the extraction yield [45].

Throughout the course of the last century, synthetic methodologies have been refined, leading to several strategies to finally synthesize CBN (**5a**) in excellent yields and solving the problem of obtaining this compound through extraction from the plant.

### 3.1. Semisynthesis

Since CBN (**5a**) is an oxidative degradation product of D**^9^**-THC (**1a**), aromatization of its natural precursor or its analogues is one of the simplest and most effective ways to finally achieve it. Different methods have been proposed for oxidizing the C ring of D^9^-THC (**1a**) and several of its regioisomers to CBN (**5a**): Adams first reported this reaction under relatively harsh conditions (heating with sulfur at approximately 250 °C) [42]. The reaction can be performed, in a less extreme environment, using *N*-bromosuccinimide and carbon tetrachloride in the presence of UV light as well, as reported by Razdan [46]. Recently, chloranyl (tetrachloro-1,4-benzoquinone) was found to selectively oxidize D^9^-THC (**1a**) while leaving other isomeric tetrahydrocannabinols unaffected [47]. Another protocol for dehydrogenation, involving selenium dioxide and trimethylsilyl polyphosphate (prepared from P_4_O_10_ and hexamethyldisiloxane), has also been described [23].

**Scheme 1:**
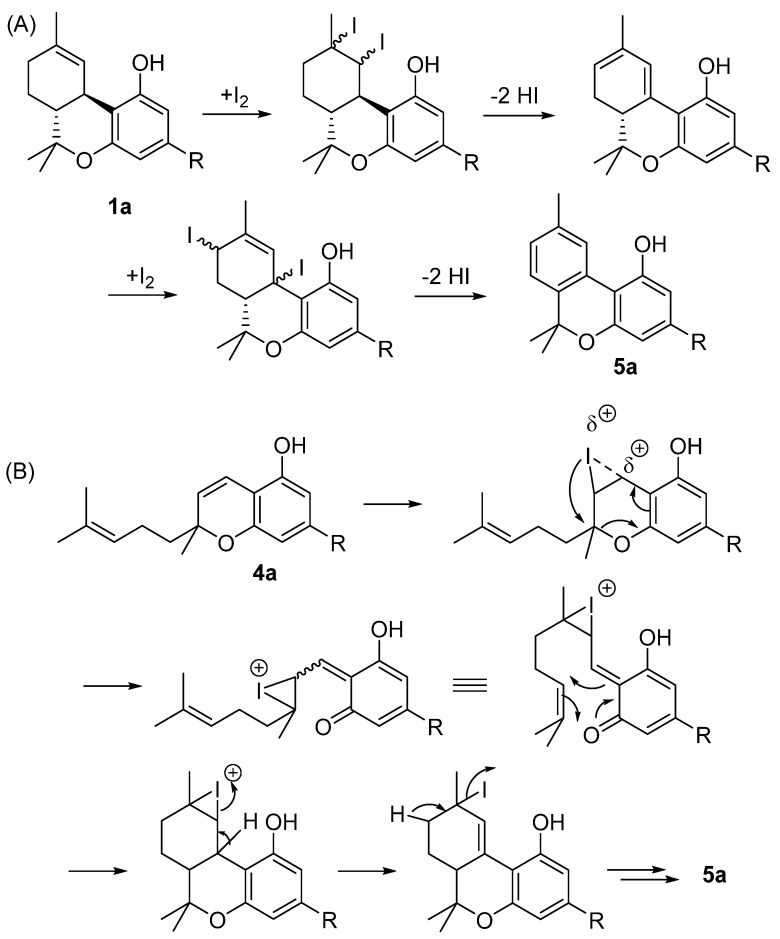
(**A**) The semisynthesis of CBN (**5a**) through an iodine-promoted cyclization of Δ^9^-THC (**1a**); (**B**) semisynthesis of CBN (**5a**) through an iodine promoted prenylchromene-benzochromane rearrangement of CBC (**4a**). R= *n*-pentyl.

We have recently reported that the iodine treatment of D^9^-THC (**1a**) and THCA (**1b**) directly produces CBN (**5a**) through a series of iodination–dehydroiodination steps driven by the transition from menthyl to *p*-cymyl aromatization (Scheme 1) [48]. Likewise, CBD (**2a**) or CBC (**4a**) can be used as starting materials, yielding between 50 and 70%. As a result of the acidic environment, CBD (**2a**) in situ cyclization occurs to D^9^-THC (**1a**), whereas with CBC (**4a**), the addition of iodine to the chromene bond causes the electrocyclic opening of the heterocyclic ring, followed by a hetero Diels–Alder reaction, which leads to tetrahydrocannabinol derivatives that are then aromatized through iodine addition-hydroiodic acid elimination reactions [49].

### 3.2. Total Synthesis through Lactonic Intermediate

**Figure 3:**
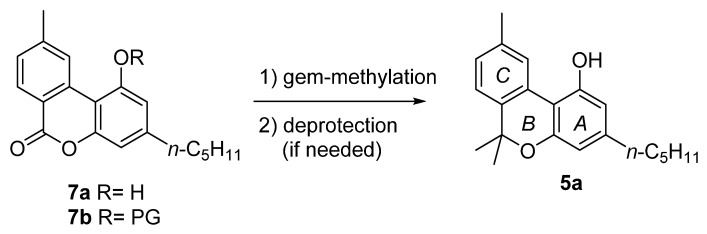
The structure of lactone intermediate **7a**–**b**.

The majority of the total synthesis of CBN (**5a**) involves the achievement of lactone intermediate **7a** or **7b** (Figure 3), which is then converted into **5a** by gem-methylation, and the subsequent deprotection of the phenolic hydroxyl, if needed.

**Scheme 2:**
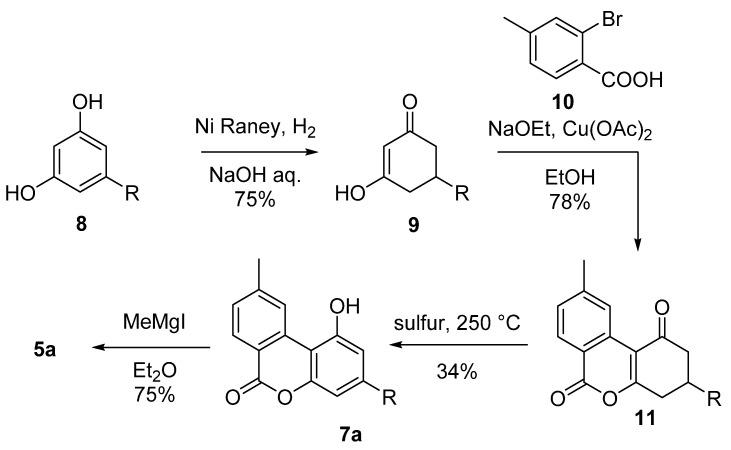
Adams’ synthesis of CBN (**5a**). R = *n*-pentyl.

This includes the first total synthesis of CBN (**5a**) conducted by Adams in 1940 in order to confirm the chemical structure of the natural product (Scheme 2) [42]. In his synthesis, Adams relied on the condensation of 5-*n*-amyl-1,3-cyclohexanedione (**9**), obtained from the hydrogenation of olivetol (**8**) with Nickel–Raney, with methyl-2-bromobenzoic acid (**10**) to form the corresponding pyrone **11**. The subsequent aromatization carried out with sulfur provides lactone **7a**, which was gem-methylated through the use of methyl magnesium iodide to finally provide **5a** with an overall moderate yield.

Nevertheless, in the most recent synthetic strategies, the aromatization step has been set aside in favour of other techniques, which allow for a greater degree of efficiency and yield to be achieved. Depending on the method by which lactone **7a** was obtained, these can be grouped into two main categories: through a biphenyl coupling and through cyclization reactions.

#### 3.2.1. Synthesis through a Biphenyl Coupling Approach

In order to complete the tricyclic structure of CBN (**5a**), one of the most common approaches is to combine two aromatic portions in a coupling reaction to connect rings A and C of the natural product, then an intramolecular reaction to complete the formation of ring B; this is probably due to the extensive amount of research and development that has been undertaken in the last decades in an attempt to optimize the synthesis of the biphenyl core, one of the most common privileged structures in medicinal chemistry [50].

**Scheme 3:**
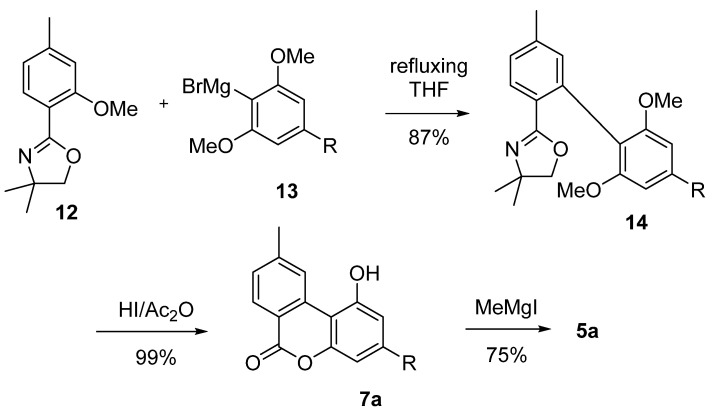
Salemink’s synthesis of CBN (**5a**) using a Grignard approach. R = *n*-pentyl.

Salemink et al. exploited Grignard’s chemistry for the coupling of the two aromatic moieties of rings A and C [51]. The Grignard’s reagent derived from 2-bromo-1,3-dimethoxy-5-pentylbenzene (**13**) was reacted with the oxazoline derivative **12** to achieve the corresponding biphenyl **14**. Next, deprotection of the phenolic hydroxyls and lactonization are obtained in a one-pot through the use of an HI/acetic anhydride mixture, which generated lactone **7a**, successively methylated to **5a** using MeMgI in accordance with Adams’ method [42] (Scheme 3). An optimized version of this approach by Miyano et al. is not based on the use of the oxazoline derivative **12**, but on its more stable and easily available 2,6-dialkylphenolic benzoate ester analogues [52].

**Scheme 4:**
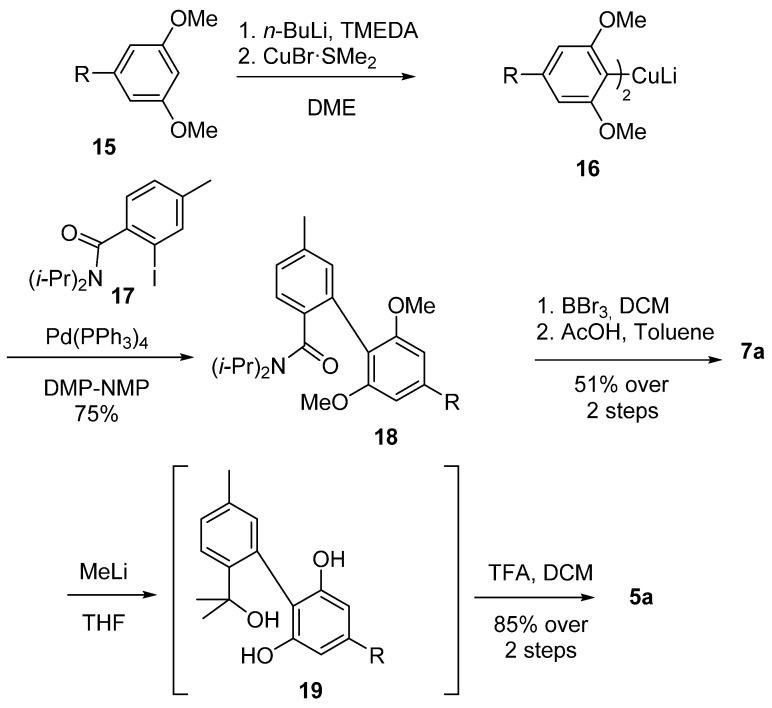
The synthesis of CBN (**5a**) according to Göttlich. R = *n*-pentyl.

Göttlich et al. synthesized the biphenyl core through a modified Ullmann–Ziegler approach using Gilman’s cuprate **16**, obtained through *ortho*-lithiation of bis-methylolivetol (**15**) with *n*-butyl lithium and a subsequent treatment with copper bromide-dimethylsulfide complex. Cuprate **16** then reacted with iodobenzamide **17** to provide the corresponding biphenyl **18** according to the Ullmann–Ziegler cross-coupling, and, after demethylation and subsequent acid-catalyzed cyclization, **7a** was achieved with 51% yield [53]. Moreover, Göttlich modified the insertion of geminal dimethyl by using two equivalents of methyllithium to afford tertiary benzylic alcohol **19**, which was then cyclized by treatment with trifluoroacetic acid to produce **5a** with a yield of 85% over two steps (Scheme 4).

**Scheme 5:**
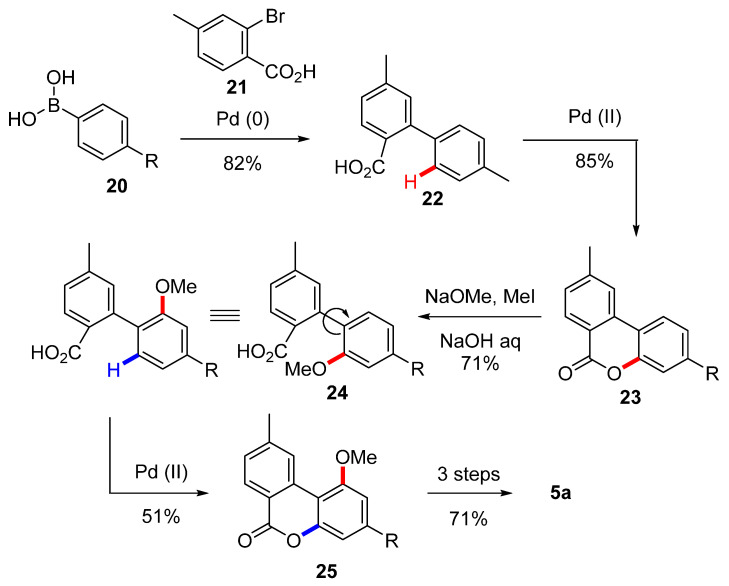
First Wang synthesis of CBN (**5a**). R = *n*-pentyl.

Wang et al. relied instead on a Suzuki coupling and then two subsequent Pd(II)/Pd(IV)-catalyzed carboxyl-directed C-H activation/C-O cyclization for the synthesis of the biaryl lactone precursor and the insertion of the phenolic oxygens [54] (Scheme 5). After a first cyclization of compound **22**, obtained by the Suzuki coupling of boronic acid **20** and bromide **21**, the corresponding lactone **23** underwent nucleophilic attack of NaOMe was then quenched with MeI. Next, hydrolysis of the ester gave the corresponding acid **24**. An additional C-H activation/C-O cyclization was conducted in order to complete assembly of the molecule’s B ring, resulting in the biphenyl lactone **25**, which was then deprotected and gem-methylated using the Göttlich’s protocol [53].

**Scheme 6:**
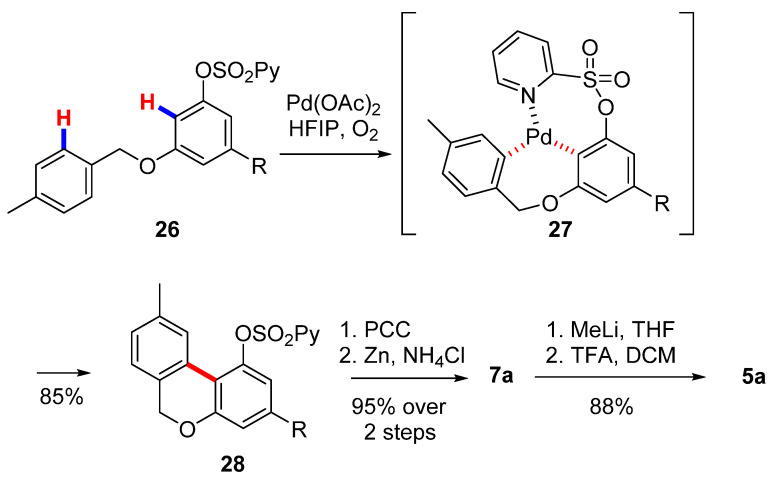
Second Wang’s synthesis of CBN (**5a**). R= *n*-pentyl.

Wang’s group also exploited an intramolecular aromatic C−H/C−H coupling catalyzed by palladium to obtain the corresponding biphenyl core [55] (Scheme 6). A sulfonylpyridyl protection on the precursor **26** is required for the coordination of palladium and activation of the C-H bond (**27**), which resulted in excellent yields of 6*H*-benzo[*c*]chromene **28**. The subsequent oxidation and deprotection of the *O*-(2-pyridyl)sulfonyl group allowed for obtaining lactone **7a**, which was been gem-methylated by means of the protocol optimized by Göttlich [53].

**Scheme 7:**
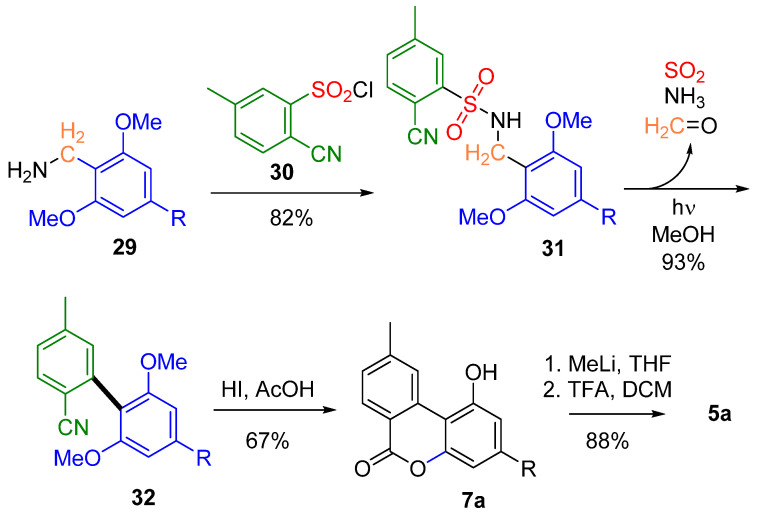
Hertweck’s metal-free CBN (**5a**) synthesis through a photosplicing reaction. R= *n*-pentyl.

The work of Hertweck et al. was instead characterized by a totally different inspiration: bypassing the use of transition metals, where a photochemical approach was used to synthesize the biphenyl portion [56] (Scheme 7). By irradiation of sulfonamide **31**, easily obtained by a nucleophilic substitution reaction involving sulfonyl chloride **30** and benzyl amine derivative **29**, the biaryl nitrile **32** was obtained, which could be simultaneously demethylated, hydrolyzed and lactonized with a yield of 67% to obtain lactone **7a**, readily converted into **5a** as previously described [55].

#### 3.2.2. Synthesis through a Cyclization Approach

In addition to the previously described methodologies, a second class of common approaches for the synthesis of biaryl lactone **7a** takes advantage of different cyclization reactions to easily build one or more cycles of the triciclyc natural product.

**Scheme 8:**
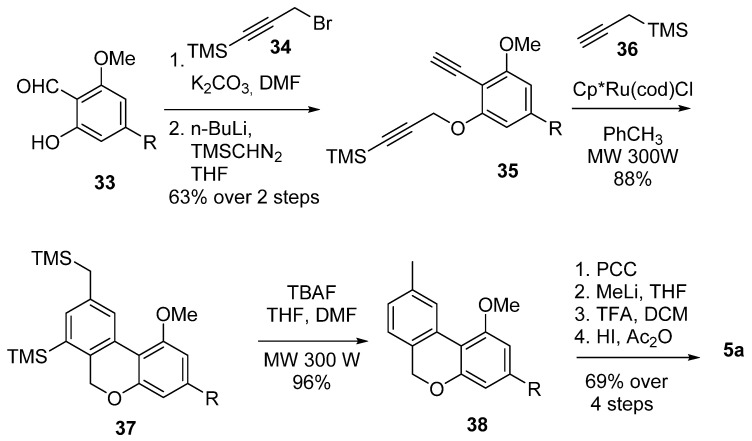
Deiters’s CBN (**5a**) synthesis through a regioselective transition-metal-catalyzed [2 + 2 + 2] cyclotrimerization. R = *n*-pentyl.

Through a regioselective [2 + 2 + 2] cyclotrimerization reaction catalysed by transition metals, Deiters et al. were able to achieve the formation of rings B and C in a single step [57] (Scheme 8): the substituted diyne **35**, obtained from salicylaldehyde derivative **33** after *O*-alkylation with **34** and reaction with TMS-diazomethane, underwent an efficient and regioselective Cp*Ru(cod)Cl catalyzed [2 + 2 + 2] cyclotrimerization reaction with propargyltrimethylsilane (**36**) under microwave irradiation, providing pyran **37** with a yield of 88% as a single regioisomer. Subsequent removal of the silyl protections using TFA afforded **38**, and its next oxidation, gem-methylation and deprotection finally resulted in **5a** with a promising yield.

**Scheme 9:**
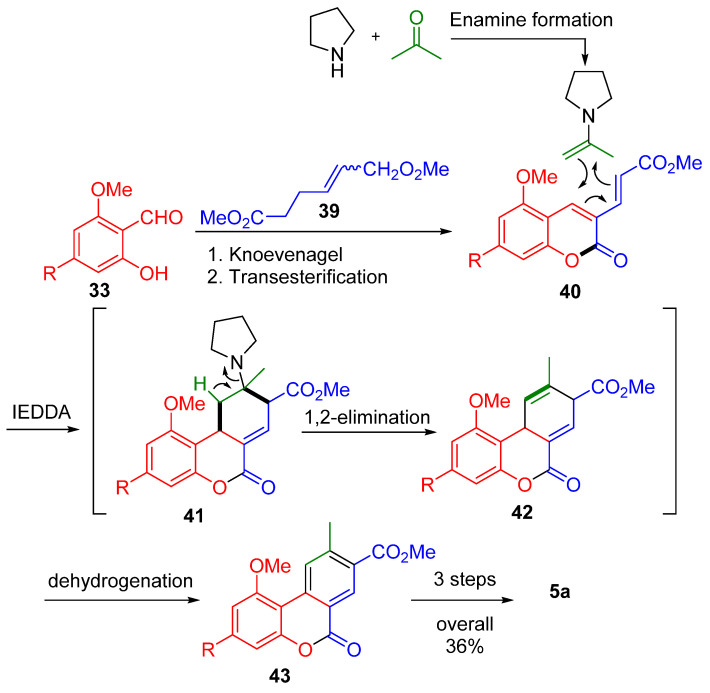
A multicomponent synthesis of CBN (**5a**) according to Bodwell. R = *n*-pentyl.

The multicomponent approach used by Bodwell et al. is also of interest, synthesizing 6*H*-dibenzo[*b*,*d*]pyran-6-ones via multicomponent domino reactions [58] (Scheme 9). The general transformation was comprised of six reactions: Knoevenagel condensation between salicylaldehyde derivative **33** and diester **39**, followed by transesterification to lactone **40**; enamine formation from acetone and pyrrolidine that then participated in an inverse electron demand Diels–Alder reaction (IEDDA) with **40**, affording the corresponding tricyclic derivative **41**, a 1,2-elimination to diene **42**, and, next, dehydrogenation to **43**. During the key inverse electron demand Diels–Alder (IEDDA) step, both diene and dienophile were generated in situ using a secondary amine.

**Scheme 10:**
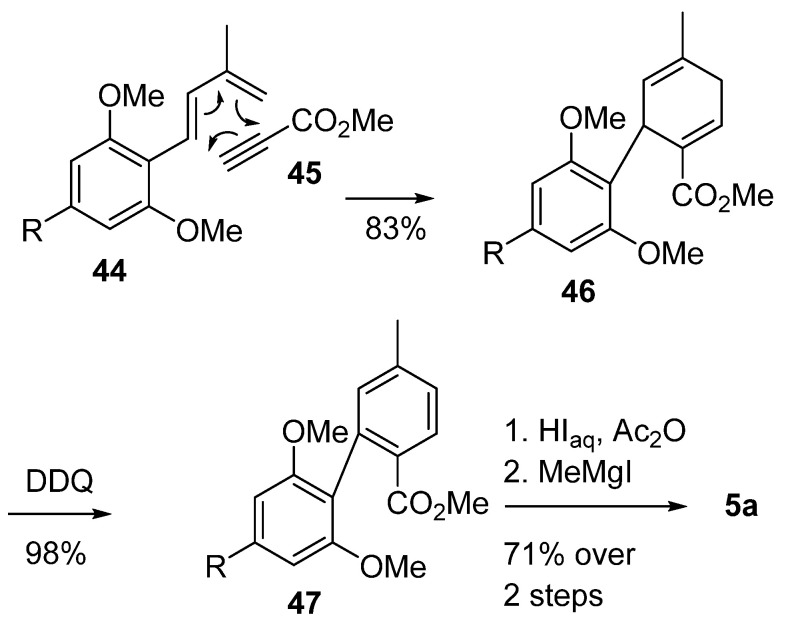
Minuti’s synthesis of CBN (**5a**) using a Diels–Alder approach. R = *n*-pentyl.

On the other hand, Minuti et al. used another Diels–Alder cyclization approach to obtain the final biphenyl **47** [59] (Scheme 10). Using olivetolic derivative **44** and methyl propiolate **45** as starting materials, phenylcyclohexadiene **46** was formed with excellent yields, and this was then aromatized with the aid of DDQ. The following steps followed what has already been reported in the literature [42].

**Scheme 11:**
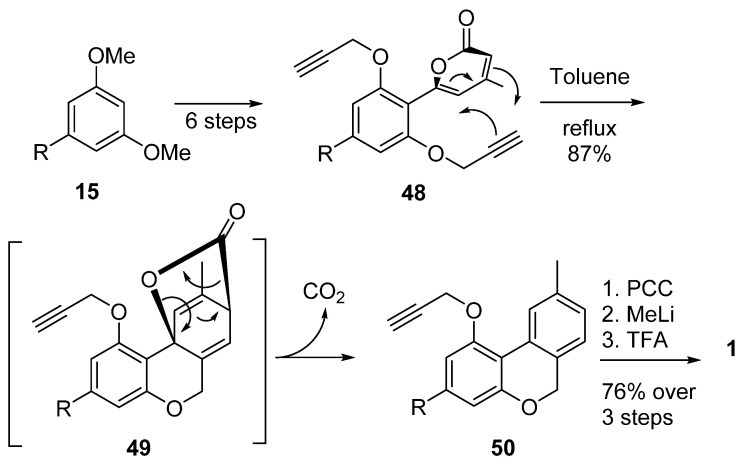
Chang’s synthesis of CBN (**5a**) by intramolecular Diels–Alder approach.

Chang et al. also used Diels–Alder chemistry, although with a different point of view: their synthesis relied upon intramolecular Diels–Alder cyclization between a pyranonic moiety and a propargyl portion (**48**), then a retro-hetero Diels–Alder (**49**) in order to obtain the 6*H*-benzo[*c*]chromene **50** with the loss of a carbon dioxide molecule [60]. After oxidation to lactone, gem-dimethylation and deprotection of the propargyl group, CBN (**5a**) was obtained with reasonable yields (Scheme 11).

**Scheme 12:**
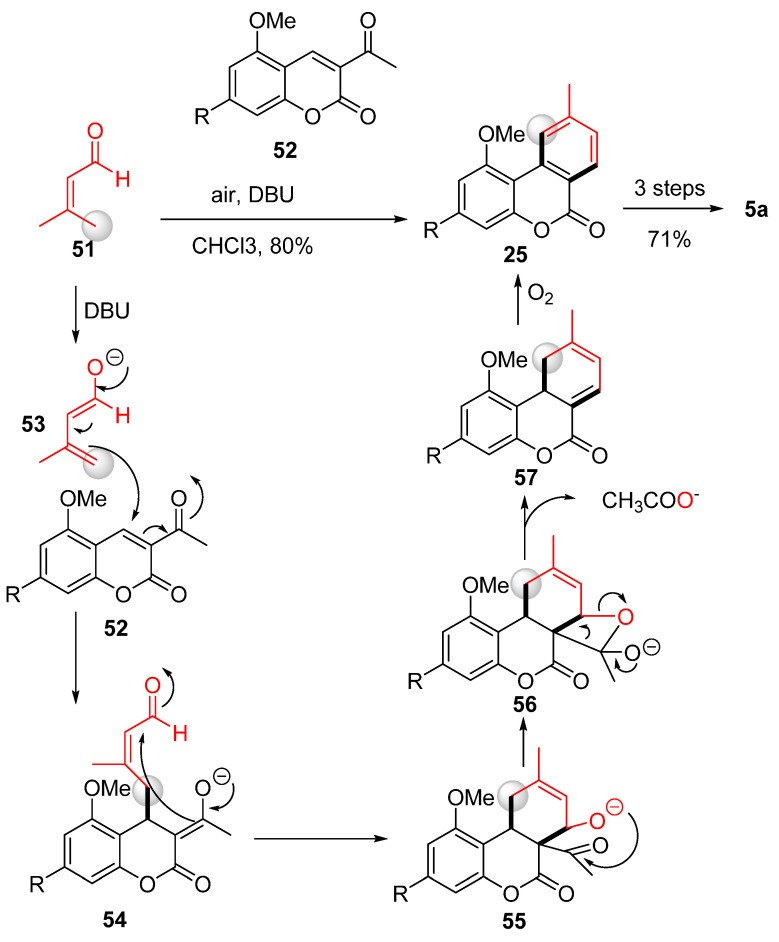
Chi’s total synthesis of CBN (**5a**). R = *n*-pentyl.

The approach taken by Chi et al. involved instead a formal [4 + 2] method to construct a new benzene ring, which enabled them to obtain different benzocoumarins, including cannabinol [61] (Scheme 12). In this process, the C ring was formed in excellent yields through the interaction between the enol form (**53**) of aldehyde **51** and the acetylcoumarin derivative **52**. The reaction proceeded via a Michael-type addition of the enal g-carbon of intermediate **53** to coumarin **52** forming intermediate **54**, which underwent an intramolecular aldol reaction to form tricyclic intermediate **55**. Following intramolecular acetal formation, **56** was obtained. As a result of the elimination of an acetate from **56**, compound **57** was formed; then, the reaction was completed via spontaneous oxidative aromatization (with air as an oxidant) to **25**. The subsequent synthetic steps followed what has already been reported [57].

### 3.3. Total Synthesis through Non-Lactonic Intermediate

The development of syntheses without the lactone intermediate **7a** is relatively rare; however, these syntheses are equally important and play a fundamental role in the background of the chemistry of CBN (**5a**).

**Scheme 13:**
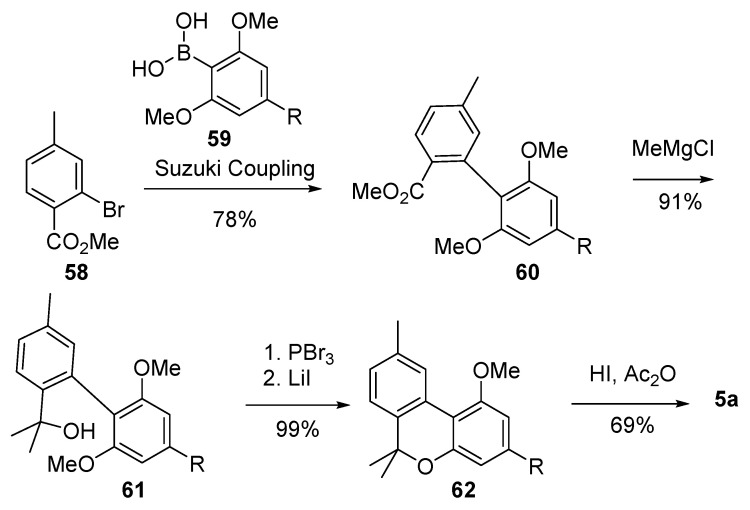
Ruchirawat’s synthesis of CBN (**5a**). R = *n*-pentyl.

As part of their studies on the synthesis of 6-alkyl-6*H*-benzo[*c*]chromenes, Ruchirawat et al. developed a one-pot cyclization/selective ether cleavage reaction which was then applied to the total synthesis of CBN (**5a**) [62] (Scheme 13). During the event, the product of Suzuki coupling (**60**) between bromide **58** and boronic acid **59** was gem-dimethylated to **61** before the formation of the B ring, obtained by treatment of the latter with PBr_3_, followed by the addition of LiI, which led to the corresponding benzo[*c*]-chromene derivative **62**. Final demethylation afforded CBN (**5a**) in good yields.

**Scheme 14:**
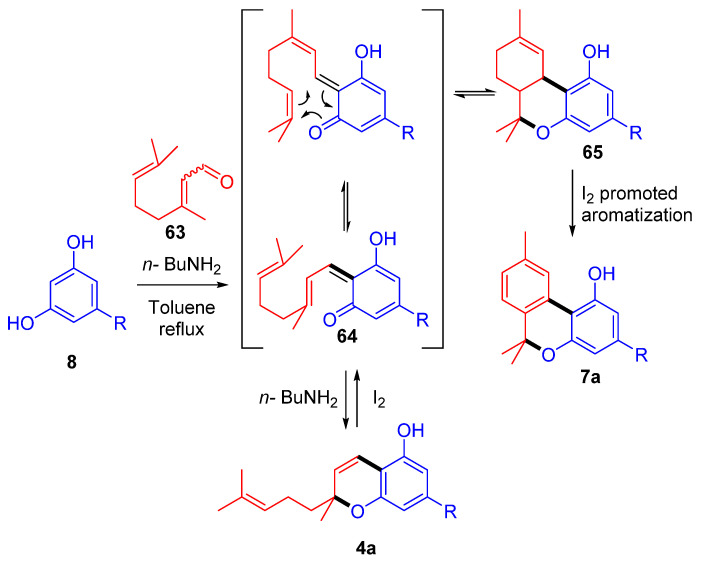
Iodine-promoted one-pot total synthesis of CBN (**5a**) using prenylchromene-benzochromane rearrangement. R = *n*-pentyl.

More recently, our research group, inspired by the reactivity studies of natural phytocannabinoids with molecular iodine [48,63], developed the first one-pot total synthesis of CBN (**5a**) from commercially available starting material exploiting an iodine-mediated deconstructive annulation [49] (Scheme 14). In the event, olivetol (**8**) and citral (**63**) reacted in basic condition to afford the corresponding homoisoprenylchromene CBC (**4a**), which, treated with iodine after removal of the amine catalyst using ionic acidic resin, underwent electroreversion and then hetero Diels–Alder cyclization through a prenylchromene-benzochromane rearrangement, affording a stereoisomeric mixture of THC derivatives **65**. The final iodine-catalyzed aromatization was a driving force strong enough to move the overall equilibrium toward the final product CBN (**5a**), afforded in an outstanding 82% yield.

## 4. Biological Profile

The ECS is an extremely important biological system and the main target of the phytocannabinoids: it modulates a wide range of cognitive and physiological processes that are necessary for regulating the body’s homeostatic state [64]. Although the mechanism by which the ECS regulates metabolism is not completely understood, it is thought that its action is largely through cannabinoid ligands activating cyclic AMP/receptor activation-related pathways [65].

The majority of the biological studies conducted on the cannabinome have focused on the “big four” phytocannabinoids; nevertheless, different studies have been conducted to elucidate CBN (**5a**) pharmacological potential, which have revealed a peculiar and interesting, although sometimes contradictory, biological profile.

### 4.1. Biochemical Assays

#### 4.1.1. Cannabinoid Receptors (CBs)

CBs are the primary and most studied targets of phytocannabinoids, as well as eponymic receptors. They belong to the G protein-coupled receptor superfamily, and currently two subtypes of cannabinoid receptors are known, namely CB1 and CB2. Generally speaking, the direct activation of CB1 can be characterized by narcotic (euphoric) effects, as well as analgesic, orexic, and anxiety-modulating activities, with the narcotic ones absent in allosteric activators. The activation of CB2 has instead anti-inflammatory and immunity-modulating properties [64]. Despite the fact that CBN (**5a**) is an agonist of both types of CBs, it exhibits different properties depending on the receptor type. The affinity of CBN (**5a**) for the CB1 receptors are 10 times lower when compared to that of D^9^-THC (**1a**) [66,67,68]; moreover, CBN (**5a**) is less effective than D^9^-THC (**1a**) at inhibiting the CB1 receptor-mediated activity of adenylyl cyclase [66]. The activity on CB2, on the other hand, has been reported to have a changeable profile from study to study; while previously CBN (**5a**) was considered an agonist with the same potency as D^9^-THC (**1a**) [69], it has recently been reported to have a potency between 2–4 times lower [66]. It is possible that the discrepancies are caused by different concentrations of CBN (**5a**) used in each experiment, as well as by the different conformational states of the receptors in the different tissues involved in the test.

As for CBN (**5a**) activity on the hypothetical CB3 receptor—the GPR55—there is very little information published, and the few available data show that it has an almost negligible effect in vitro [70].

#### 4.1.2. Transient Receptor Potential Channels (TRPs)

Several types of animal cells contain TRP channels, which are ionic channels located primarily on their plasma membranes. Various chemical and physical stimuli (as an example, heat and cold somatosensation) are transduced by these membrane proteins, and pain signals are also generated by them. They are also capable of causing and sustaining inflammation as a result of their activation [71].

Several types of TRPs are modulated by CBN (**5a**): it acts as an agonist at TRPV1, TRPV2, TRPV3 and TRPV4 channels, stimulating the Ca^2+^ influx, as well as activating Ca^2+^-dependent pathways in the cells [71]. Regarding other TRP variants, CBN (**5a**) inhibits the icilin-induced activation of TRPM8 (also known as the cold and menthol receptor 1, CMR1) by acting as a potent antagonist [71]. The natural compound has also been demonstrated to stimulate the TRPA1 channel in a potent and effective manner, exhibiting an EC_50_ value of 0.18 ± 0.02 mmol [71].

### 4.2. Pre-Clinical and Clinical Assays

It does not appear that CBN (**5a**) has been extensively investigated preclinically or clinically (no human pharmacokinetic and metabolism data have been reported), although the published results indicate that CBN (**5a**) has a promising pharmacological profile. Despite the positive results of the studies that have been published so far, the results are often still open to doubt, since a large number of the research did not test the most suitable pathological endpoints and did not achieve the outcomes that were expected.

#### 4.2.1. Analgesic and Anti-Inflammatory Activity

As an analgesic and anti-inflammatory agent, CBN (**5a**) has been found to be potentially useful in the treatment of pain. Previous researches have reported that CBN (**5a**) can mitigate the symptoms of myofascial pain disorders, including temporomandibular disorders and fibromyalgia in rats by reducing the mechanical sensitivity induced by intramuscular injections of nerve growth factor in the masseter muscles [72], with an increasing activity when administered in a 1:1 mixture with CBD (**2a**). CBN (**5a**) could attenuate the production of interleukins 2, 4, 5, 13 and decrease allergen mucus production in OVA-sensitized and challenged A/J mice, classifying it as a potential treatment for allergic airway diseases [73]. The use of CBN (**5a**) for the treatment of glaucoma was also suggested since it prevents inflammation which causes elevated intraocular pressure [74]. Moreover, preliminary studies have demonstrated that CBN acts as an antioxidant and decreases cell damage in a cell culture model of Huntington disease [75].

#### 4.2.2. Antibacterial Activity

In the same way that other cannabinoids have been shown to be effective against a number of antibiotic-resistant bacteria, e.g., CBG (**3a**) and CBC (**4a**), CBN (**5a**) also appears to have antibacterial properties; it has been proven to be highly effective against multiple bacteria that are resistant to antibiotics, including methicillin-resistant Staphylococcus aureus (MRSA), making it a potentially effective treatment for staph infections [76].

#### 4.2.3. Orexigenic Activity

As well as stimulating hyperphagia, CBN (**5a**) increases food consumption and feeding time in rats [77,78]. However, since CBN (**5a**) is not a narcotic, it may have greater potential as an orexic drug in conditions where the stimulation of appetite is beneficial, such as terminal cancer and HIV infection.

#### 4.2.4. Treatment of Epidermolysis Bullosa

The use of CBN (**5a**) has recently been shown to be effective in the treatment of epidermolysis bullosa, a group of rare medical conditions characterized by easy blistering of the skin and mucous membranes. Phase 2 studies undertaken by InMed Pharmaceuticals are currently in progress [79] as a follow-up of the previous Phase 1 studies conducted by the same, showing that the CBN (**5a**) based preparation in development was safe and well-tolerated on induced open epidermal wounds, causing no systemic or serious adverse effects [80].

#### 4.2.5. Sleep Induction

As already shown in Section 2, high concentrations of CBN (**5a**) are found in aged cannabis products, since it is the main degradation product of D^9^-THC (**1a**). As a result of unclear reasons, CBN (**5a**) has been associated with the induction of sleep, causing the cannabinoid to be marketed under the name “the sleepy cannabinoid in old weed” at dosages generally less than 5 mg/die [81]. Despite this, sleep studies involving CBN (**5a**) have not reached a clear conclusion as to whether this claim is true.

CBN (**5a**) was reported to increase barbiturate-induced sleep time in one study [82], but this result was not replicated in disaccordance with a previous study [83]. Nevertheless, as shown in a murine study [84], CBN (**5a**), combined with D^9^-THC (**1a**), increased sedation synergistically, and this observation was reproduced in a small clinical trial involving five male volunteers who received oral CBN (50 mg), D^9^-THC (12.5 mg), or two different combinations of them (12.5 mg Δ^9^-THC + 25 mg CBN or 25 mg D^9^-THC and 50 mg CBN), separated by a one-week washout period [84]. As well as pain threshold and skin sensitivity, which are not affected by CBN alone, the combination did not alter the effects of D^9^-THC (**1a**) on heart, blood pressure, and body temperature [84]. Nonetheless, the combination led to modest increases in some subjective outcomes (drowsiness and dizziness). It is therefore unsubstantiated that CBN (**5a**) has sleep-inducing properties, from a clinical perspective [85].

## 5. Conclusions

The studies reviewed demonstrate the significant contribution that CBN (**5a**) has made to the development of knowledge pertaining, from chemical and biological perspectives, to the field of cannabinoids, even if in a sometimes indirect and particular manner. Since the beginning of the chemistry associated with the study of this class of compounds, CBN (**5a**) has played a crucial role, actively participating in the clarification of their biological properties, the elucidation of the structure of the major cannabinoids, and the development of innovative and efficient extraction and purification processes.

As a result of the subsequent isolation of the major cannabinoids, particularly CBD (**2a**) and ∆^9^-THC (**1a**), and the discovery of the remarkable biological activity associated with them, CBN (**5a**) has unfortunately often been overlooked in favor of its analogues which demonstrate a more marked and promising biological profile; however, it has recently regained prominence following the discovery of the ECS and the multitude of targets that comprise it and provide new avenues for the study and treatment of a broad spectrum of diseases. Despite the large number of biological studies conducted on this compound, further research is necessary: extensive data on pharmacokinetics and pharmacodynamics are required, especially on larger mammals, as well as a complete screening on the large number of secondary targets correlated to the ECS.

The peculiar biphenyl structure of CBN (**5a**) has made it the target compound for the validation of numerous and diversified synthetic techniques, keeping it in high regard from the 1940s to the present day, and a number of important synthetic methodologies have been developed through the study of its chemistry, including a straightforward one-pot protocol for its synthesis from commercially available source materials.

The current simplicity of synthesis, both of CBN (**5a**) and its analogues, combined with the largely unknown biological properties, make this class of benzo[c]-chromenic structured compounds one of the most powerful platforms for exploration of the biological space associated with the chemotype of cannabinoids.

## Data Availability

Not applicable.
